# Reviewed article: Gabe KT, Junior GB, Constante J. Adaptation and validation of a tool for assessing food knowledge based on the Nova classification for the Brazilian context. Epidemiol Serv Saude. 2025:34;e20240335.

**DOI:** 10.1590/S2237-96222024v34e20240335.a

**Published:** 2025-05-02

**Authors:** Clareci Silva Cardoso

**Affiliations:** Universidade Federal de São João del-Rei

**Table:** 

Rating	Excellent	Good	Average	Below average	Poor
Interest	✓				
Quality		✓			
Originality	✓				
Overall		✓			

**Table:** 

Questionnaire	Yes	No	Not applicable
Does the manuscript contain new and significant information to justify publication?	✓		
Does the Abstract (Summary) clearly and accurately describe the content of the article?		✓	
Is the problem significant and concisely stated?	✓		
Are the methods described comprehensively?		✓	
Are the interpretations and conclusions justified by the results?	✓		
Is adequate reference made to other work in the field?	✓		

## Manuscript Structure:

Length of article is: Adequate

Number of tables is: Adequate

Number of figures is: Adequate

Please state any conflict(s) of interest that you have in relation to the review of this paper (state “none” if this is not applicable): None

## Comments

Trata-se de um tema relevante para o contexto da Epidemiologia e Serviços de Saúde, porém sugere que o artigo seja reformulado e submetido para reavaliação, para possível publicação.

Segue arquivo com sugestões em anexo

